# Bufalin Alters Gene Expressions Associated DNA Damage, Cell Cycle, and Apoptosis in Human Lung Cancer NCI-H460 Cells *in Vitro*

**DOI:** 10.3390/molecules19056047

**Published:** 2014-05-13

**Authors:** Shin-Hwar Wu, Yung-Ting Hsiao, Jaw-Chyum Chen, Ju-Hwa Lin, Shu-Chun Hsu, Te-Chun Hsia, Su-Tso Yang, Wu-Huei Hsu, Jing-Gung Chung

**Affiliations:** 1 Institute of Clinical Medical Science, China Medical University, Taichung 40402, Taiwan; 2 Division of Critical Care Medicine, Department of Medicine, Changhua Christian Hospital 50006, Taiwan; 3 Department of Biological Science and Technology, China Medical University, Taichung 40402, Taiwan; 4 Department of Medicinal Botany and Health Applications, Da-Yeh University, Changhua 51591, Taiwan; 5 Gradualted Institute of Chinese Medical Science, China Medical University, Taichung 40402, Taiwan; 6 Department of Internal Medicine, China Medical University Hospital, Taichung 40447, Taiwan; 7 Department of Radiology, China Medical University Hospital, Taichung 40447, Taiwan; 8 Department of Internal Medicine, China Medical University, Taichung 40402, Taiwan; 9 Department of Biotechnology, Asia University, Taichung 41354, Taiwan

**Keywords:** bufalin, cDNA microarray, DNA damage, cell cycle, apoptosis, NCI-H460 cells

## Abstract

Lung cancer is the leading cause of cancer related death and there is no effective treatment to date. Bufalin has been shown effective in inducing apoptosis and DNA damage in lung cancer cells. However, the genetic mechanisms underlying these actions have not been elucidated yet. Cultured NCI-H460 cells were treated with or without 2 μM of bufalin for 24 h. The total RNA was extracted from each treatment for cDNA synthesis and labeling, microarray hybridization, and then followed by flour-labeled cDNA hybridized on chip. The localized concentrations of fluorescent molecules were detected and quantitated and analyzed by Expression Console software (Affymetrix) with default RMA parameters. The key genes involved and their possible interaction pathways were mapped by GeneGo software. About 165 apoptosis-related genes were affected. CASP9 was up-regulated by 5.51 fold and THAP1 by 2.75-fold while CCAR1 was down-regulated by 2.24 fold. 107 genes related to DNA damage/repair were affected. MDC1 was down-regulated by 2.22-fold, DDIT4 by 2.52 fold while GADD45B up-regulated by 3.72 fold. 201 genes related to cell cycles were affected. CCPG1 was down-regulated by 2.11 fold and CDCA7L by 2.71 fold. Many genes about apoptosis, cell cycle regulation and DNA repair are changed significantly following bufalin treatment in NCI-H460 cells. These changes provide an in depth understanding of cytotoxic mechanism of bufalin in genetic level and also offer many potentially useful biomarkers for diagnosis and treatment of lung cancer in future.

## 1. Introduction

Non-small cell lung carcinoma is one of leading causes of cancer-related death in the United States and throughout the world [1]. It causes more than one million deaths every year [2]. Most non-small cell lung cancer patients are diagnosed at late stages (stage IIIb or IV) and are inoperable. Standard platinum-based chemotherapies provide marginal improvement in survival at the expense of substantial toxicities [3]. Even with the addition of target-therapy, the median survival of metastatic non-small cell lung cancer patients is about one year [4]. Because of the unsatisfactory results of standard chemotherapy, many advocate finding new drugs.

Natural products were the main source of health care in ancient times. In modern medicine, they are still major sources of new drug development. Chan Su is derived from the serous fluid of the posterior auricular glands of toad. Its anti-tumor effects have been reported in many Chinese treatises. Bufalin is thought to be the key component of Chan Su. Modern researches also confirm bufalin-induced cytotoxic effects on many human cancer cells [5,6,7,8,9]. The mechanisms of action of bufalin on cancer cell have been shown to include apoptosis-induction [10], interruption of damaged DNA repair [11], halting uncontrolled cell cycle [12] and inhibition of cancer cell migration/metastasis. 

Genetic mutations play a pivotal role in oncogenesis. Virtually all human cancers have mutations interfering cell cycle checkpoints and leading to uncontrolled cell growth. Some oncogenic mutations involving inactivating apoptosis or disrupting DNA repair mechanism. These vulnerable genes are thus targets of detection or treatment for various cancers. 

There is little research exploring the mechanisms of bufalin’s anti-cancer effects at a genetic level. By Northern blot or RT-PCR, these studies focused on less than five genes at one time [6,13,14]. There have been no studies using cDNA microarrays for extensive genetic surveys for this purpose till now. In this study, we used cDNA microarrays to investigate the genetic change of NCI-H460 lung cancer cells following bufalin treatment *in vitro*. We put special emphases on genes related to cell cycle regulation, apoptosis induction and DNA repair. Through this study, we are closer to understand the mechanisms underlying bufalin’s anti-cancer effects at the genetic level. We hope this knowledge will provide a basis for future studies on anti-cancer drug development or modification. 

## 2. Results and Discussion

### 2.1. The Up-Regulated and Down-Regulated Gene Expression in H460 Exposed to Bufalin

H460 cells were treated with or without 2 μM of bufalin in 12 well-plates for 24 h, then cells were harvested and total RNA were extracted and measurement of concentration and then cDNA microarray analysis of gene expression was performed. The calculated gene expressions from microarrays are shown in Table 1. Table 1 indicates that six genes were over 20-fold and 21 were over 10-fold up-regulated. Eleven genes were down-regulated over 10-fold and 42 genes over 6-fold. Table 2 shows the descriptions of genes highly influenced by bufalin treatment. Among those affected genes, 165 are associated with apoptosis, such as CASP9 (caspase 9, apoptosis-related cysteine peptidase) which was up-regulated 5.51-fold. THAP1 (THAP domain containing, apoptosis associated protein 1) was up-regulated by 2.75-fold. CCAR1 (cell division cycle and apoptosis regulator 1) was down-regulated by 2.24-fold. One hundred and seven affected genes are associated with DNA damage and repair, such as MDC1 (mediator of DNA-damage checkpoint 1), that was down-regulated by 2.22-fold or DDIT4 (DNA-damage-inducible transcript 4) which was suppressed by 2.52-fold, whereas, GADD45B (growth arrest and DNA-damage-inducible, beta) was up-regulated by 3.72-old. As for genes related to cell cycle regulation, 201 were affected. For example, CCPG1 (cell cycle progression 1) was down-regulated by 2.11-fold and CDCA7L (cell division cycle associated 7-like) was inhibited by 2.71-fold.

**Table 1:** Number of genes by the fold change after bufalin treatment.

Fold Change	Number of Genes
≥20	6
≥10 and <20	21
≥5 and <10	59
≥4 and <5	53
≥3 and <4	118
≥2 and <3	488
>−3 and ≤−2	1215
>−4 and ≤−3	348
>−5 and ≤−4	99
>−6 and ≤−5	57
>−10 and ≤−6	42
<−10	11

#### 2.2. GeneGo Analysis Program from Bufalin Treated H460 Cells Demonstrated the Top Alteration in Gene Expression Scored by the Number of Pathway Networks

**Figure 1:**
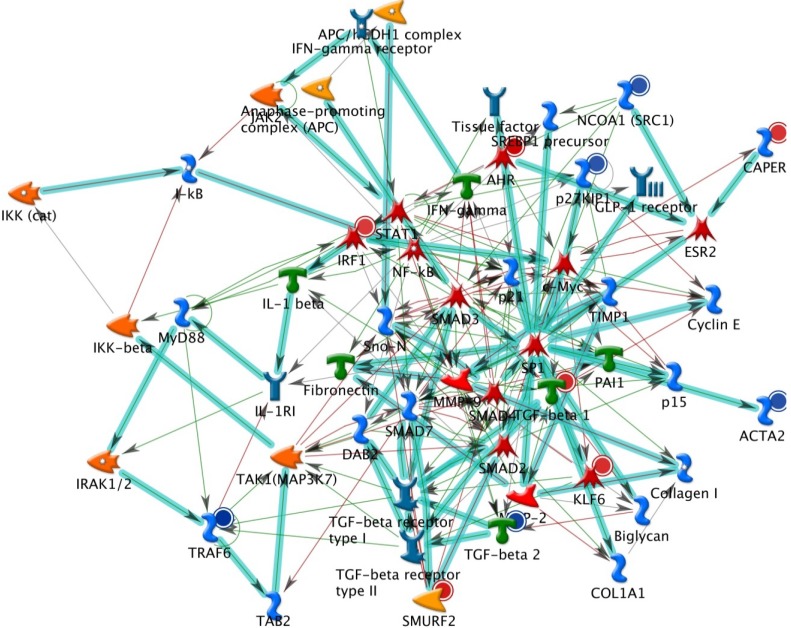
The top scored (by the number of pathways) AN network from GeneGo 02. Thick cyan lines indicate the fragments of canonical pathways. Red circles represent up regulated genes and down regulated with blue circles represents down-regulated genes. The ‘checkerboard’ color indicates mixed expressions for the genes between files or between multiple tags for the same gene.

**Figure 2:**
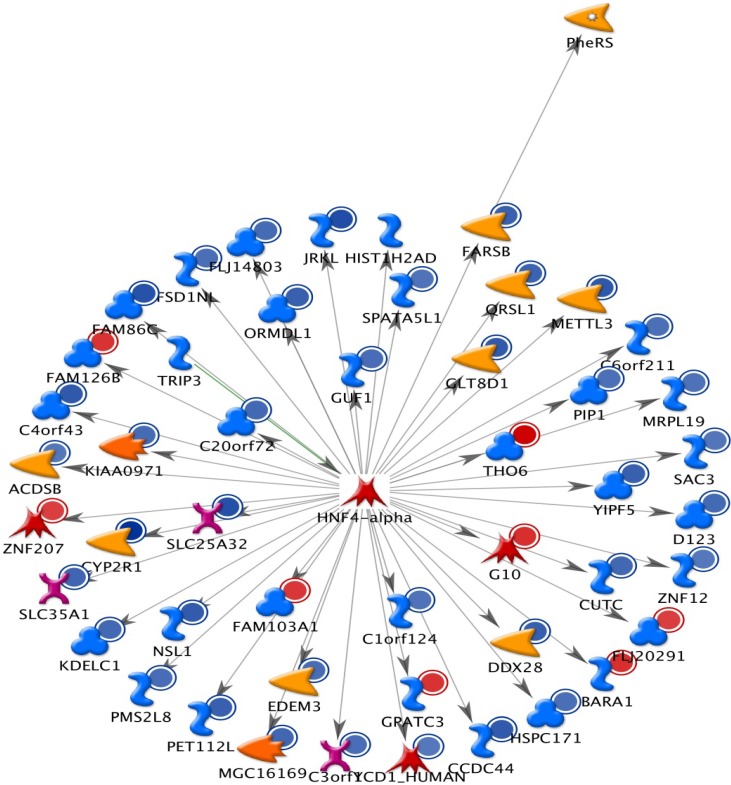
The second scored (by the number of pathways) AN network from GeneGo 02. Thick cyan lines indicate the fragments of canonical pathways. Red circles represent up regulated genes and blue circles represent down-regulated genes. The ‘checkerboard’ color indicates mixed expressions for the genes between files or between multiple tags for the same gene.

**Figure 3:**
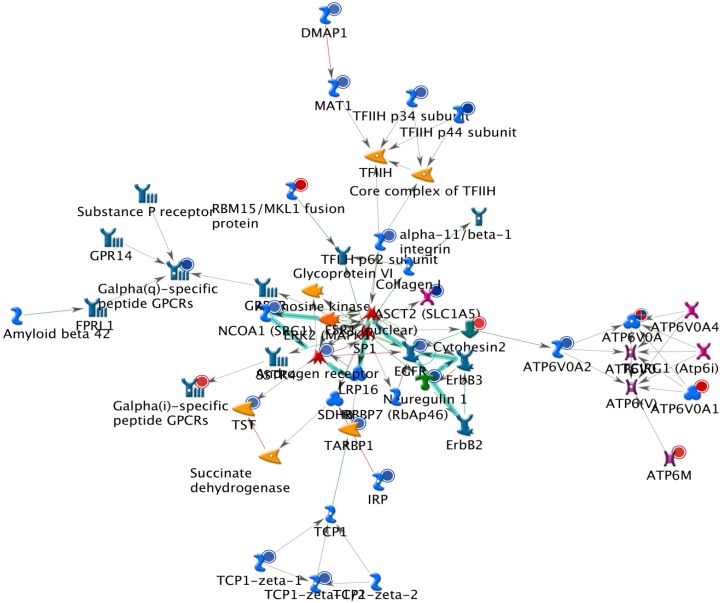
The third scored (by the number of pathways) AN network from GeneGo 02. Thick cyan lines indicate the fragments of canonical pathways. Red circles represent up regulated genes and blue circles represent down-regulated genes. The ‘checkerboard’ color indicates mixed expressions for the genes between files or between multiple tags for the same gene.

The results from GeneGo analysis are shown in Figure 1, Figure 2 and Figure 3. Experimental data were mapped on the processes and shown as red (up‑regulation) and blue (down‑regulation) circles of different intensities indicating different inhibitions in NCI-H460 cell after bufalin treatment. 

**Table 2:** Representative genes of NCI-H460 were influenced by bufalin.

Fold change	Gene Symbol	mRNA Description
66.33	EGR1	early growth response 1
26.16	EGR2	GTP binding protein overexpressed in skeletal muscle
24.09	EGR3	pyruvate dehydrogenase kinase, isozyme 4
23.48	EGR4	NOTCH-regulated ankyrin repeat protein
23.10	EGR5	histone cluster 1, H4d
20.63	EGR6	FBJ murine osteosarcoma viral oncogene homolog
5.52	BTG	BTG family, member 2
5.51	CASP9	caspase 9, apoptosis-related cysteine peptidase
4.54	CRY2	cryptochrome 2 (photolyase-like)
4.36	AEN	apoptosis enhancing nuclease
3.72	GADD45B	growth arrest and DNA-damage-inducible, beta
2.75	THAP1	THAP domain containing, apoptosis associated protein 1
2.02	FLJ43315	similar to Asparagine synthetase [glutamine-hydrolyzing] (Glutamine-dependent asparagine synthetase) (TS11 cell cycle control protein)
−2.11	CCPG1	cell cycle progression 1
−2.22	MDC1	mediator of DNA-damage checkpoint 1
−2.24	CCAR1	cell division cycle and apoptosis regulator 1
−2.25	SMC3	structural maintenance of chromosomes 3
−2.52	DDIT4	DNA-damage-inducible transcript 4
−2.71	CDCA7L	cell division cycle associated 7-like
−3.18	DCLRE1C	DNA cross-link repair 1C (PSO2 homolog, S. cerevisiae)
−3.93	UIMC1	ubiquitin interaction motif containing 1
−5.04	DCLRE1A	DNA cross-link repair 1A (PSO2 homolog, S. cerevisiae)
−10.55	CCND2	cyclin D2
−10.57	C5orf33	chromosome 5 open reading frame 33
−11.08	GPR65	G protein-coupled receptor 65
−11.38	PRICKLE1	prickle homolog 1 (Drosophila)
−11.93	ORC5L	origin recognition complex, subunit 5-like (yeast)
−12.02	AHNAK2	AHNAK nucleoprotein 2
−12.24	ICT1	immature colon carcinoma transcript 1
−12.61	KRT19	keratin 19
−15.54	OCLN	occludin
−27.38	MIR1977	microRNA 1977

### 2.3. Discussion

In this study, we found the gene AEN of NCI-H460 cells was up-regulated by 4.36-fold after bufalin treatment. AEN encodes a protein called ‘apoptosis-enhancing nuclease’, which is usually located in the nucleolus and is translocated to the nucleoplasm upon stimulation by apoptosis signals. Ionizing radiation or DNA-damaging agents such as adriamycin could induce phosphorylation of Ser-15 of apoptosis-enhancing nuclease by p53 and lead to the activation of the latter. Upon activation, apoptosis-enhancing nuclease exerts an exonuclease function and cleaves double-stranded DNA into single-stranded one to augments p53-induced apoptosis [15,16].

Bufalin treatment also led to a 3.72-fold increase of the gene GADD45B, which is a member of a group of genes usually up-regulated following stressful growth arrest or DNA damage [17]. These genes regulate cell growth and apoptosis by mediating activation of the p38/JNK pathway via their proteins binding and activation of upstream activator MTK1/MEKK4 kinase. These genes often cooperate with different mechanisms to inhibit cell growth [18]. GADD45B has been found to have potential clinical usefulness as a diagnostic or grading parameter for malignancies [19]. 

The gene DCLRE1A, also known as SNM1A, was down-regulated by 5.04-fold in NCI-H460 cells treated with bufalin. DCLRE1A encodes the DNA cross-link repair 1A protein (hSNM1A), which is located in the nuclei of cells from various tissues such as brain, heart, kidney, liver, pancreas, placenta and skeletal muscle [20]. This protein belongs to the DNA repair metallo-β-lactamase family and is involved in DNA inter-strand cross-link repair. It is also required for checkpoint mediated cell cycle arrest in early prophase following mitotic spindle stress [21].

Cyclin D2 is associated with and activates the catalytic activities of cyclin-dependent kinase (CDK) 4 and CDK6 to facilitate cell cycle transition from G1 to S in mammalian cells. One of the most significant substrates of cyclin D2-CDK complexes is Rb protein, a tumor suppressor protein known for its association with hereditary retinoblastoma. CCND2, the gene encoding cyclin D2, has been found related to several human malignancies including glioblastoma [22], leukemia [23] and ovarian cancers. Over-expression of CCND2 is associated with increased invasiveness in human squamous cell carcinoma *in vivo* [24]. We found CCND2 gene was down-regulated by 10.55-fold in NCI-H460 cells treated with bufalin. This substantial suppression may contribute to the cytotoxic and anti-metastasis effects of bufalin on NCI-H460 cells. 

Because many genes related to apoptosis, cell cycle regulation and DNA repair are changed following bufalin treatment; we present their complex interactions in Figure 1, Figure 2 and Figure 3. These figures just represent our understanding based on current knowledge and assumptions. They are not meant tobe comprehensive, nor undebatable. More studies are needed to expand our current understanding.

## 3. Experimental

### 3.1. Chemicals and Reagents

Bufalin and dimethyl sulfoxide (DMSO) were purchased from Sigma Chemical Co. (St. Louis, MO, USA). Culture medium RPMI-1640 with additional 10% fetal bovine serum and 1% l-glutamine were purchased from Gibco BRL (Grand Island, NY, USA). Bufalin was dissolved in DMSO and stocked at 20 °C.

### 3.2. Lung Cancer Cells

The NCI-H460 human lung cancer cell line was purchased from the Food Industry Research and Development Institute (Hsinchu, Taiwan). It was maintained in a RPMI-1640 medium plus 10% fetal bovine serum, 1% l-glutamine, 100 units/mL of penicillin G and 100 microgram/mL of streptomycin. The cells were kept in a 37 °C incubator under 5% CO_2_ and 95% air. The cells were sub-cultured at 80%–90% confluency [25,26,27].

### 3.3. cDNA Microarray Assay for Genes Expression in H460 Cells after Exposure to Bufalin

NCI-H460 cells were placed at a density of 5 × 10^5^ cells/mL in RPMI 1640 medium for 24 h. Cells were treated without (control) and with 2 μM of bufalin for 24 h. The total RNAs were extracted by Qiagen RNeasy Mini Kit (Qiagen, Inc, Valencia, CA, USA) and were used for cDNA synthesis and labeling, microarray hybridization, and then followed by flour-labeled cDNA hybridizing their complements on the chip (Affymetrix GeneChip Human Gene 1.0 ST array, Affymetrix, Santa Clara, CA, USA). Also, the resulting localized concentrations of fluorescent molecules were detected and quantified (Asia BioInnovations Corporation, Taipei, Taiwan). Finally, the resulting data were analyzed by Expression Console software (Affymetrix) with default RMA parameters [28,29,30,31]. Genes regulated by bufalin by at least a 2-fold change were recorded. Data are representative of three separate assays. 

## 4. Conclusions

In conclusion, we have demonstrated in this study that many genes connected with apoptosis, cell cycle regulation and DNA repair are changed significantly following bufalin treatment. These changes provide an in-depth understanding of the mechanism(s) of bufalin cytotoxicity in a genetic level and also offer many potentially useful biomarkers for diagnosis and treatment of lung cancer.
